# The British Nurses' Association: An Argument and a Warning

**Published:** 1889-07-06

**Authors:** A. Ernest Sansom

**Affiliations:** Physician to the London Hospital


### The British Nurses' Association: An Argument and a Warning.

By A. Ernest Sansom, M.D., F.R.C.P., Physician to the London Hospital.

Dr. Sansom published the following statement on the subject of the registration of nurses in last week's *British Medical Journal:*

"As it appears to me that the proposals of the British Nurses' Association have not received sufficient consideration from the medical profession, and as in my opinion these proposals involve questions of high public policy, I ask you to afford me some space in which to develop my views.

"When the association was first started I was privileged to be present at one of the meetings, and I ventured to offer some suggestions as to the proposed rules. With the object of the association as stated in the draft by-laws ('to unite British nurses for their mutual help and protection, and for the advancement in every way of their professional work') I had the most heartfelt sympathy.

"Those who attempted to organise the undertaking obtained the support of ladies and gentlemen of the highest possible social influence, as well as of known and highly respected members of the medical profession, many of them my own personal friends. There can be no question of the good intentions of these; many of them would, however, admit that they had no very decided experience in the training of nurses, and some perhaps have not fully considered the proposals of the organisers of the association as these became gradually developed. For myself, when I came to comprehend and consider the general scheme, whilst approving its object, I felt that I could not identify myself with its methods.

"Now that some of those who have published arguments in opposition to the scheme are treated with the severest forms of criticism, their motives impugned, and the opprobium heaped upon them extended over the great institution to which it is my privilege to belong, I feel that I should no longer keep silence, but avow myself as sharing the opinions of those who are so roughly handled, and—though not without protest—submit to my fair proportion of the stripes wherewith they are castigated.

"I have already quoted the printed statement of the objects of the British Nurses' Association. I have some right to express myself in sympathy with this. I took great interest in the institution of the Nursing Home for the London Hospital, and I continued for four years to direct the course of instruction so far as medical nursing is concerned. My interest in its welfare will never cease.

"To establish an association the membership of which should constitute a bond of union among nurses was, I felt sure, a laudable work. It seemed to me that the object would be best attained by the establishment of a society with a central home where matters for the good of nurses and nursing could be considered and discussed by the nurses themselves, with district branches in various parts of the country, the society to possess some literary organ to provide the means for free intercommunication. When, however, the proposal formulated by the organisers of the British Nurses' Association was announced to be that of the establishment of a general register for nurses analogous to the *Medical Register*, with a charter of incorporation for the association, the ground became changed, and the situation required the gravest consideration. Such consideration has been given to it by those who are entitled by experience to speak—by Mr. Henry Bonham-Carter, secretary to the Nightingale Fund ('Is a General Register for Nurses Desirable?'), and by Miss Lückes, matron of the London Hospital ('What will Trained Nurses Gain by Joining the British Nurses' Association?'). With the opinions enunciated by Mr. Bonham-Carter and Miss Lückes I am in the main agreed, and, after mature consideration, I feel it right to express my opinion that the scheme of the British Nurses' Association, so far as it is now intelligible, is fraught with peril to the best interests of the public, the medical profession, and the nurses themselves. I will divide my remarks into two sections: 1. The question of training; 2. The question of diploma.

"1. *Training.*—The by-law (iv.) of the British Nurses' Association provides that all who have been engaged during three years in nursing in any public hospital or infirmary, or public or district nursing association in the British Empire, all trained midwives and women who have been engaged in private nursing, shall be eligible as members (that is, for registration and the diploma) on producing to the executive committee satisfactory evidence of professional attainments and personal character. It is obvious therefore, that whilst some candidates will be adequately trained, others cannot be considered to be trained at all. What is the guarantee of training of the private nurse who has pursued her vocation at Little Peddlington for three years? and why, after the obtaining of her diploma, should she not pursue a persistent course of wrong-doing? I think that most people would infer from the phrase that the applicants must produce to the executive committee 'evidence of professional attainments' that some sort of examination was contemplated, but it would appear from the *Nursing Record* of June 13, 1889, that nothing on this point has been yet determined.

"There are few people, I think, who would deny that the systematic training of a nurse is desirable. At the London Hospital Training School for Nurses a term of two years' practical instruction in the wards is required of the probationer. During this period she must attend a course of twelve lectures by the matron on the general details of nursing, followed by sixteen lectures by Mr. Treves on elementary anatomy and surgical nursing, and sixteen lectures by Dr. James Anderson on elementary physiology and medical nursing. At the expiration of the term she must present herself for an examination, theoretical and practical, on the subjects in which she has been taught.

"I would not exaggerate the importance of the examination. At any rate, it seems to weed out those who remain persistently ignorant or are culpably careless, whilst it gives valuable indications concerning the qualities of diligence and precision. It is not always, however, that those who pass best at the examinations are the best nurses. Such tests necessarily fail to indicate the virtues of gentleness, sympathy, and tact, which make a nurse's services most acceptable to the sick. In the list of those who have gone through the period of probation, and have successfully passed the examination, are nurses possessed of various qualities and capabilities. A matron who has to recommend a nurse for certain duties in public or private situations will from her personal knowledge be able to select the one she considers best suited to the individual case.

"I do not assert, nor do I believe, that the system of training at the London Hospital is better than that which obtains in many institutions in London and in the country; but it must be very obvious that such would be impossible except in the case of he minority of those qualified by the by-law of the British Nurses' Association to become members of that corporation. It is proposed, therefore, to give the trained and the untrained a similar diploma—a process of levelling-down which appears to me to be very undesirable. The answer to Miss Lückes's question, 'What will trained nurses gain by joining the British Nurses' Association?' seems at the very outset obvious.

"I now turn to the second branch of the subject under consideration.

"2. *The Diploma.*—It is contemplated to obtain for the British Nurses' Association a Royal charter enabling it 'to grant a certificate and to confer on those whom it regards as qualified to undertake the work of nursing a status which no unauthorised person will be able to usurp.' In fact, a register analogous to the *Medical Register* is to be instituted. Let us enquire whether such a step is justified on the ground of public policy. It can scarcely be contended that the question of registration of doctors and registration of nurses are similar. Such evidence of qualification is obviously important in the case of the medical practitioner who has to fulfil well-defined duties to the State; in return the State allows the recipient of the diploma to hold certain public offices, and gives him the privilege (denied to the advertising quacks and irregular practitioners, who, nevertheless, seem to thrive wondrously well) to sue for fees.

"But how stands the question as regards the nurse? Let me put it practically. When a sick nurse is required in a private family, what is the usual course of action? I hope and believe the medical attendant is consulted? He is probably acquainted with the qualities and capabilities of certain nurses, or he sends to an institution which he believes to confer adequate instruction and training. If such institutions are as yet insufficient, let efforts be made to multiply, as well as strengthen them. Is there no danger that under the scheme which we are considering, the cordial relations which should subsist between patient and doctor will be disturbed? Is it improbable that the patient will, in the first instance, consult the register and choose the nurse? Let my brethren in general practice carefully consider this question. Further, is there no danger that if a nurse should become endowed with a diploma, a still more harmful competition may ensue? It is our general experience that the best trained nurse presumes the least. She learns to know how much she does not know, and the difficulties and the responsibilities of the qualified practitioner of medicine are understood and appreciated by her. Not all, however, are in this category, for in the nurse's walk of life, as in others,

"'Fools rush in where angels fear to tread.'

A nurse with a legal diploma is scarcely the same person as the nurse the watchful servant of the patient, and the obedient and trusted assistant of the doctor.

"There are many among the public who would take no very particular trouble to investigate the difference of significance between the diplomas, and the step might be a very easy one between the administration of medicinal means and the advising as well as advertising them. Some may consider that this prospect is visionary, but let my colleagues in rural districts and in the populous parts of cities duly consider the situation.

"At any rate, I crave that the questions involved be discussed without fear and without reproach.

"To my understanding, the pamphlets of Mr. Bonham-Carter and Miss Lückes are in the language and the spirit of fair criticism.

"Whether the expressions contained in the *Nursing Record* of June 13, 1889, which calls itself 'the representative organ of the nursing profession,' can be so characterised, I must ask those who are interested in the questions involved to judge.

"In conclusion, I must in fairness state that the views I have just enunciated are my own, expressed without any consultation with my colleagues interested in the Nursing Home of the London Hospital."

